# Proteolysis dependent cell cycle regulation in *Caulobacter crescentus*

**DOI:** 10.1186/s13008-022-00078-z

**Published:** 2022-04-01

**Authors:** Nida I Fatima, Khalid Majid Fazili, Nowsheen Hamid Bhat

**Affiliations:** 1 Department of Biotechnology, University of Kashmir, Hazratbal, Srinagar, Jammu and Kashmir 190006 India; 2 Department of Biotechnology, Central University of Kashmir, Ganderbal, Jammu and Kashmir 191201 India

**Keywords:** Cell cycle progression, Regulatory proteolysis, Cell division proteins, Molecular medicine, Translational health

## Abstract

*Caulobacter crescentus*, a Gram-negative alpha-proteobacterium, has surfaced as a powerful model system for unraveling molecular networks that control the bacterial cell cycle. A straightforward synchronization protocol and existence of many well-defined developmental markers has allowed the identification of various molecular circuits that control the underlying differentiation processes executed at the level of transcription, translation, protein localization and dynamic proteolysis. The oligomeric AAA+ protease ClpXP is a well-characterized example of an enzyme that exerts post-translational control over a number of pathways. Also, the proteolytic pathways of its candidate proteins are reported to play significant roles in regulating cell cycle and protein quality control. A detailed evaluation of the impact of its proteolysis on various regulatory networks of the cell has uncovered various significant cellular roles of this protease in *C. crescentus*. A deeper insight into the effects of regulatory proteolysis with emphasis on cell cycle progression could shed light on how cells respond to environmental cues and implement developmental switches. Perturbation of this network of molecular machines is also associated with diseases such as bacterial infections. Thus, research holds immense implications in clinical translation and health, representing a promising area for clinical advances in the diagnosis, therapeutics and prognosis.

## Introduction

Targeted protein degradation is a universal phenomenon in all living cells. Predominantly governed by energy dependent proteases in prokaryotes, regulatory proteolysis is indispensable for cellular viability and environment sensing pathways [1]. The past decade has seen resurgence in understanding of bacterial development, which once was thought to be preserve of eukaryotic systems [2]. Akin to eukaryotic multicellular organisms, bacterial cell systems are highly organized with exquisitely dynamic regulatory mechanisms that control the morphological and functional programs on a spatio-temporal scale [3, 4]. Exploiting *Caulobacter crescentus*, an experimentally tractable model organism as a genetic tool, the research progress thus far has elucidated fundamental insights into protein degradation, a process important for normal cell biology. In *C. crescentus,* ClpXP protease is critical for driving cell cycle progression. The levels of a number of proteins that are involved in cell division are controlled by this protease. Therefore, a comprehensive understanding of the impact of its proteolysis on various regulatory networks of the cell have revealed novel cellular roles of this protease in *Caulobacter* leading to identification of research areas that will accelerate understanding protease biology [5]. Detection of the panel of different players of regulatory proteolysis and how they work at the molecular level, reveal an insight in implicating their predictive therapeutic significance. The present review is aimed to discuss the progress in understanding the multifaceted regulatory strategies of proteome dynamics and its implications in molecular medicine for targeted therapeutics and molecular diagnostics.

### *Caulobacter crescentus* divides asymmetrically to yield two different progeny cells

**Fig. 1:**
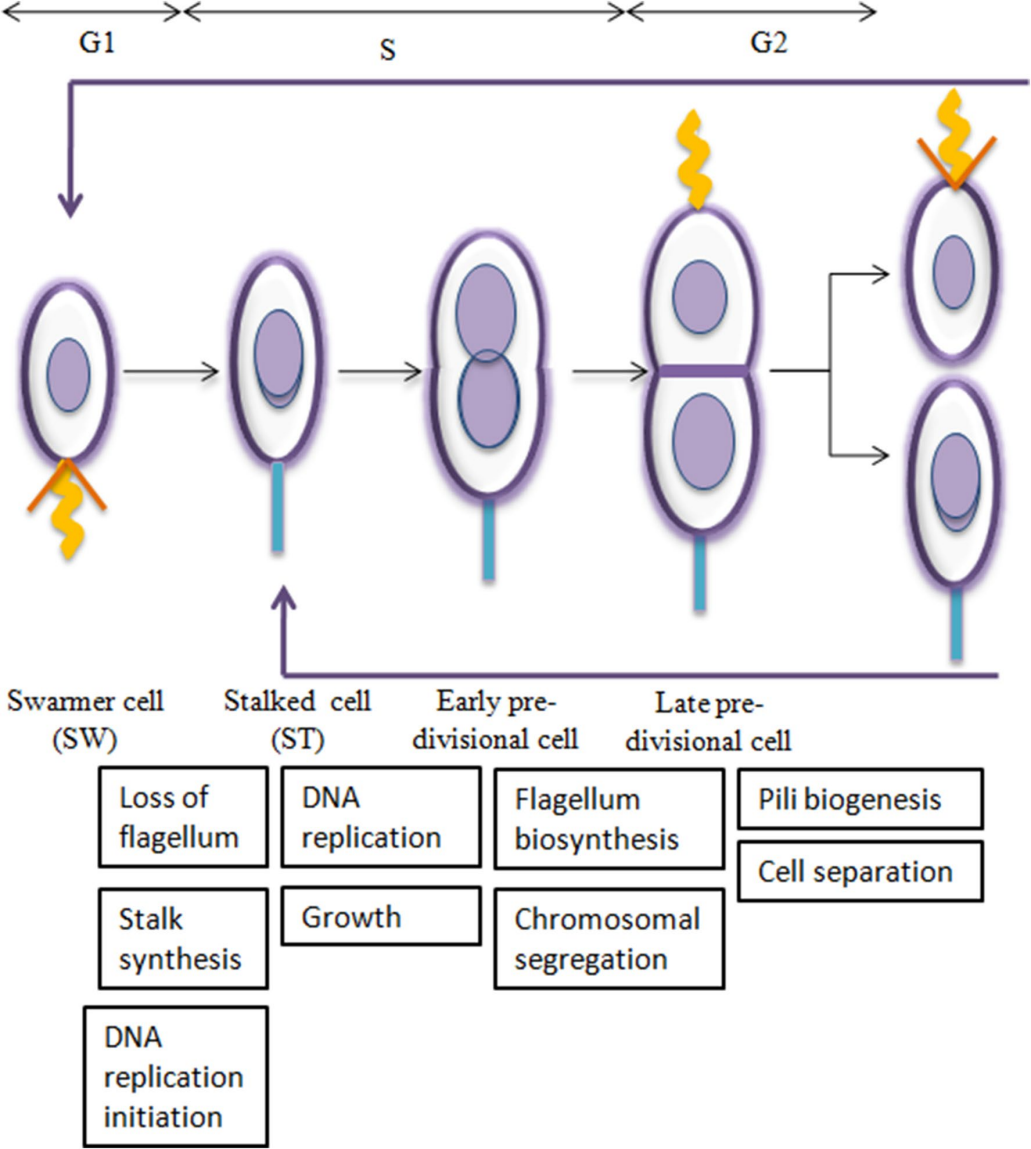
*Caulobacter* cell cycle: each cell divides asymmetrically into two distinct cells with different morphologies and fate. Double arrows above indicate the phase of cell cycle corresponding to the morphology and boxes below show the events as the cell progresses through cell cycle

A hallmark of *Caulobacter* is a characteristically defined cell cycle, leading to a dimorphic life cycle. It undergoes a simple developmental program within each cell cycle resulting in formation of two different daughter cells, each having identical genome but different morphology and fate [6–9]*.* This is unlike the widely studied model microorganism, *E. coli* that produce similar progeny on division [10]. The life cycle starts with a motile chemotactic swarmer cell that bears single polar flagellum and polar type IV pili, aiding in motility for exploring the environment for resources and adhesion to surfaces respectively. The flagellum along with the chemosensory apparatus mediates the directional movement in a gradient of attractants or repellents. The swarmer cell is unable to start DNA replication and continues to remain in G1 phase till its morphological transition into another cell type, the stalked cell (Fig. 1). When the proper environmental conditions are met, swarmer cell discards its polar flagellum and retracts the pili to differentiate into non-motile stalked cell. A new stalk, which helps in adhesion to solid surfaces, is produced by elongation of cell wall and membranes at the site of discarded flagellum. Coincident with this swarmer- to stalked cell transition, the cell enters S phase and initiates the new round of DNA replication.

Akin to most eukaryotes and distinct from prokaryotes such as *E.coli,* the DNA replication in *Caulobacter* occurs exactly once per cell cycle [11, 12]. It originates at a single origin and proceeds bi-directionally [13]. Being a fundamental event in cell cycle, the initiation of DNA replication is tightly regulated at many levels [14]. Partitioning of chromosomes begin simultaneously and continues through most of the cell cycle [15, 16]. The newly synthesized chromosomes move towards poles by pushing and pulling mechanisms in *Caulobacter* which is unlike *E.coli* where such mechanisms are absent [17]. A new flagellum is built in the late predivisional cell at the pole opposite to stalk, before its division into two morphologically and physiologically different daughter cells. Significantly, a straightforward synchronization of morphologically and developmentally distinct populations has enabled uncovering details of widely conserved mechanisms of DNA replication and cell cycle regulation, less tractable in symmetrically dividing model species such as *E. coli.* The cell cycle progression relies on the oscillating level of many proteins during which a swarmer cell develops into a stalked cell, followed by an asymmetric cell division. Stalked cell immediately initiates another round of replication undergoing growth and cell division, whereas the swarmer cell that enters into G1 phase and remains in that phase until the obligate swarmer to stalked differentiation step.

### Cell cycle in *Caulobacter* is tightly regulated at spatio-temporal levels

Regulation of the cell cycle is required for proper growth and development of all organisms. The developmental events in bacteria are tightly intertwined with the cell cycle [18]. Like eukaryotes, bacteria have complex regulatory mechanisms that synchronize the cell cycle progression with growth and development [19]. This includes the spatio-temporal control of DNA replication and cell division to ensure that the dividing cells get equal number of chromosomes. A cell-type-specific gene expression profile is observed in the sequential series of morphogenetic and physiological changes with the progression of cell cycle of *C. crescentus*. Expression of about 20% of all *Caulobacter* genes varies during the cell cycle [20]. Transcription of cell cycle regulated genes peaks with the cellular process requiring that gene product. Another process that regulates the levels of different protein, as the cell progresses through cell cycle, is time specific degradation [21]. Proteolysis of many proteins [5, 22], replication factors [23], metabolic enzymes [24, 25] as well as some chemoreceptors [26] is required for cell to progress through cell cycle. There is a precise balance between protein synthesis and degradation such that the protein levels are maintained in a steady-state. Regulation of either synthesis or degradation, or both, generates a change in concentration of many proteins throughout the cell cycle [27]. While the fluctuation in levels of some protein arises due to the cyclic change in synthesis coupled with the constitutive degradation; in other proteins, there is change in degradation rate during progression of cell cycle with the synthesis remaining constitutive. In some proteins of *Caulobacter*, dynamics is controlled by both transcriptional regulation of synthesis and post-translational control of degradation in a cell cycle dependent manner. A detailed evaluation of regulatory proteolysis of candidate proteins is aimed by analyzing spatiotemporal tracking of gene expression, protein subcellular localization, chromosome segregation and growth over the course of cell cycle [28]. Diverse cellular and molecular processes needed to duplicate a cell, such as DNA replication and accurate segregation of chromosomes to daughter cells, are characteristically aberrant in many conditions. Therefore, understanding this regulation is crucial to the study of several diseases.

### Regulatory proteolysis plays an essential role in cell cycle progression

Regulated protein degradation is a universal phenomenon. Studies, so far, have shown proteolysis to play a crucial role in development and cell cycle progression. In prokaryotes, highly conserved ATP-dependent proteases are deployed for regulated degradation and protein quality control [29]. Bacteria, including *Caulobacter*, use a number of energy dependent AAA+ proteases for controlled destruction of proteins. Most of the AAA + proteases are made up of two conserved functional domains [30]. ATPase module recognizes the protein, either directly or through adapters, and uses energy from hydrolysis of ATP to fuel unfolding of protein. The unfolded protein then encounters the peptidase domain where it is destroyed.

The selectivity of substrate is determined by ATPase/unfoldase part of the protease possessing intrinsic capacity to identify the target protein [31]. For instance, ClpXP, a well-known protease in *Caulobacter*, strictly recognizes the non-polar C-terminal residues of protein and a single mutation in recognition site can render the protein non-degradable [32]. Similarly, Lon protease, another well characterized protease in *C. Crescentus* primarily involved in stress and damage response such as proteotoxic stress by heat*,* targets the poorly folded polypeptides with long stretches of exposed hydrophobic residues [33–35]. Additional specificity in proteolysis is provided by the auxiliary proteins, called adapters that alter the specificity and promote or inhibit the protease activity. These adapters either activate the protease or transport the substrates to already active protease. The activation of protease by one adaptor leads to the binding of another adaptor and so on with each level in hierarchy responsible for proteolytic destruction of different group of proteins [36, 37]. In addition to control at substrate level, a feedback mechanism has been seen to control the levels of protease itself, wherein different proteases collaborate to control the levels of one particular protease [38]. For instance, recent studies show proteolytic degradation of Lon by active Lon and ClpAP protease on exposure of its native C-terminus. This degradation of Lon by Lon itself ensures tight control of protease in order to support normal growth and simultaneously assuring an adequate capacity to handle stressful conditions.

### CLPXP is an essential protease in *Caulobacter* crescentus

Unlike *E.coli*, ClpXP protease is indispensable in *Caulobacter* [21]. The recent progress in elucidation of the multifaceted complex regulatory strategies by this essential molecular machine has led to a deeper understanding of the effect of regulatory proteolysis with an emphasis on cell cycle progression. ClpXP consists of ClpX (unfoldase) domain and ClpP (peptidase) domain [31]. It is required for turnover of a number of regulatory and other proteins (Table 1). For example, it regulates the levels of CtrA, an inhibitor of G1-S transition, in a cell cycle dependent manner. CtrA is a response regulator involved in controlling the transcription of multiple cell cycle genes. It has been described as a master regulator of cell cycle with key function involving repression of chromosome replication by binding to five origins of replication in swarmer cell [39]. ClpXP specifically degrades CtrA leading to morphological differentiation of swarmer cell into stalked cell in this organism. Cells lacking ClpXP are arrested in G1 phase and are unable to initiate chromosome replication [21]. Similarly, before the onset of S-phase, ClpXP proteolytically degrades PleC and cckN, phosphatases that keeps the levels of phosphorylated DivK (DivK*-*P) and PleD (PleD-P) low in G1-phase [40]. DivK-P and PleD-P are involved in inactivation of CtrA during G1-S transition, ensuring a multi-level regulation of developmental program. At the same time, ClpXP degrades PdeA, a phosphodiesterase required for cleavage of global secondary messenger, cyclic di-guanylic acid (cdG) [41] thus antagonizing the activity of diguanylate cyclase DgcB. The differentiation and cell cycle progression in *Caulobacter* is coordinated with phosphorylation of key regulator proteins which is in turn interlinked with secondary messenger cdG [42]. The accumulation of PdeA in swarmer cells blocks differentiation. During G1-S transition, PdeA is proteolyzed by ClpXP allowing DgcB function to unleash. DgcB together with activated PleD promote the morphogenesis. In a similar manner, the levels of developmental regulator TacA, are controlled by ClpXP in a cell cycle dependent manner (mam). TacA, σ54-dependent activator, promotes transcription of genes required for cell cycle regulated stalk biosynthesis in collaboration with RpoN [43].

**Table 1:** List of substrates degraded during cell cycle by ClpXP protease

Substrate	Role	Reference
CtrA	Replication initiation inhibitor and transcriptional regulator	21–38
PleC	Phosphatase	40
CckN	Phosphatase	40
TacA	Transcriptional regulator	43
PdeA	Cyclic di-GMP phosphodiesterase	42
McpA	Transmembrane chemoreceptor	26
McpB	Cytoplasmic chemoreceptor	46
CpdR	Single-domain response regulator/adaptor	47
RcdA	Regulator of CtrA degradation	57
GdhZ	NAD-dependent glutamate dehydrogenase	24
KidO	NAD(H)-binding oxidoreductase homolog	25
TipF	Flagellar regulator	48
NstA	Negative switch for Topo IV decatenation activity	49
MopJ	Single-domain PAS (Per-Arnt-Sim) protein	50
FliF	MS ring protein	51
FtsZ	Cell division cytoskeletal protein	66, 69
FtsA	Cell division protein	74
Trx1	Oxidoreductase	52

The essentiality of ClpXP is attributed to its important role in destruction of SocB protein. SocAB forms a non-canonical toxin-antitoxin system in *Caulobacter* [44]. Normally ClpXP binds to adaptor SocA and degrades the toxin SocB, an inhibitor of DNA replication that is upregulated during DNA damage. Cells lacking SocB toxin due to gene mutation can survive in absence of ClpXP albeit with defective morphology and growth, thus suppressing the essentiality of the protease. In addition, ClpXP, in *Caulobacter*, is essential for cell cycle progression for partially processing replication clamp loader complex, DnaX to (Table 2) produce fragments that are stable and important for responding to DNA damaging conditions such that disrupting this partial proteolysis leads to abnormal growth [45].

**Fig. 2:**
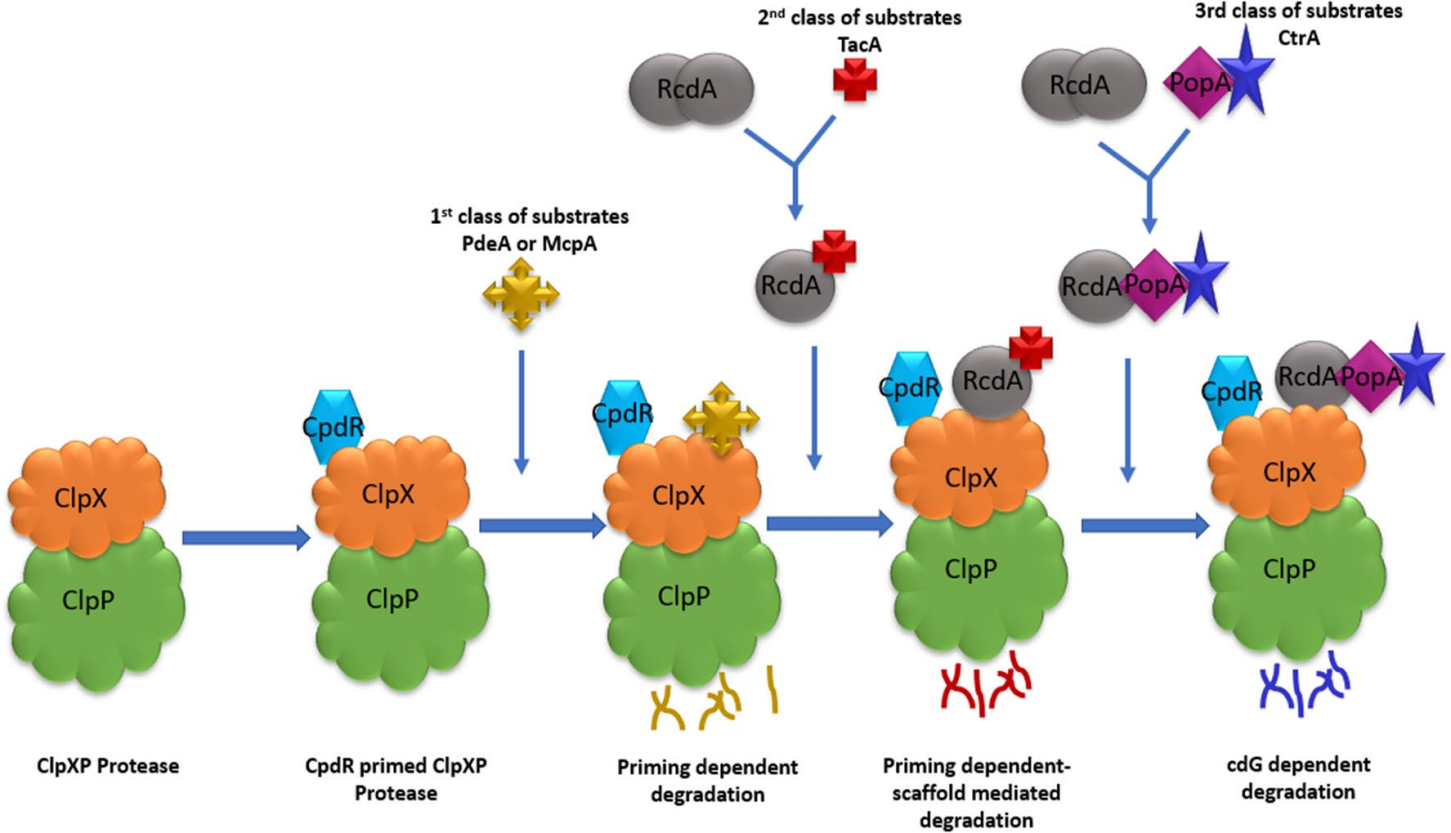
Adaptor hierarchy: adaptors assemble in hierarchical manner to degrade proteins by ClpXP protease during cell cycle progression. Adaptor CpdR primes the protease for degradation of first class of substrates such as PdeA or McpA. The primed protease can the proteolyze second class of substrates such as TacA that are bound to scaffolding adaptor RcdA. Lastly, adaptor PopA binds to RcdA to bring third class of substrates such as CtrA to ClpXP for degradation

ClpXP uses a number of adaptors to modulate substrate specificity (Fig. 2). The adaptor acts as a scaffold to tether the substrate to protease. CpdR, RcdA and PopA form a hierarchy of adaptors to deliver different class of proteins to ClpXP for destruction during progression of cell cycle in *C. crescentus* (Table 2) [36, 37]. CpdR and RcdA are conserved in all known α-proteobacteria, PopA, however is found only in *Caulobacter* and related species [53, 54]. The hierarchy begins with CpdR, a response regulator that is required for destruction of all known ClpXP substrates during cell cycle [36, 37]. In unphosphorylated state, CpdR binds to ClpXP at N-terminal of ClpX and localizes it to cell pole. In swarmer cells, CpdR is phosphorylated by CckA kinase, thus preventing ClpXP localization and therefore CtrA proteolysis. During G1-S transition, unphosphorylated CpdR accumulates to allow ClpXP localization and CtrA degradation. In addition to CtrA, CpdR is essential for destruction of all other proteins required for G1-S transition. Such first class of substrates, such as PdeA and McpA only need CpdR adaptor for ClpXP activity [42–55]. However, some protein needs additional adaptors, RcdA and PopA, or RcdA alone for degradation by ClpXP. RcdA, initially identified as factor responsible for CtrA localization, acts as a scaffolding adaptor [56]. It directly binds to second class of substrates such as TacA and delivers them to CpdR primed ClpXP [36]. The priming of ClpXP by CpdR is essential for recruitment of substrates by RcdA to the protease. RcdA is itself a target of ClpXP when it is not bound to the substrate. Recent studies have shown substrate binding competes with dimerization of RcdA for a common interface. Absence substate leads to homodimerization of adaptor. Dimeric adaptor is susceptible to degradation by protease. However, presence of substrate and its binding to RcdA relieves this dimerization and monomeric RcdA is resistant to degradation [57]. In addition to second class of ClpXP substrates, RcdA has binding affinity for another adaptor PopA, a cdG-binding effector protein, essential for CtrA proteolysis by ClpXP(R). PopA binds to RcdA via its N-terminal while CtrA binding to PopA in cdG-dependent fashion results in dimerization of adaptor at C-terminal [54]. However, it was recently seen that PopA is present in distinct oligomeric states at the two poles. At stalked pole it is present in dimeric form while at swarmer pole it appears in monomeric form due to asymmetrically distributed cdG [58]. In its dimeric state, PopA acts as an adaptor for ClpXP to control levels of third class of substrates and in monomeric form, along with its binding partner, SmrF, regulates the length of flagella filament. Thus, asymmetric distribution of cdG at two poles during cell cycle provides a spatiotemporal cue to asymmetric division of cell.

**Table 2:** An adaptor hierarchy regulates proteolysis during a *Caulobacter crescentus* cell cycle

Adaptor	1st class substrate	2nd class substrate	3rd class substrate
CpdR	✔	✔	✔
RcdA	✖	✔	✔
PopA	✖	✖	✔

### Lon contributes to proteolytic regulation of cell cycle proteins

Apart from ClpXP, another protease that is involved in cell cycle regulation of some of the proteins is Lon protease (Table 3). Cells lacking Lon are viable but show low growth and defects in motility, chromosome content and division. Akin to CtrA degradation by ClpXP, another cell cycle regulator, SciP is degraded by Lon during G1-S transition. SciP is small inhibitor of CtrA that accumulates in G1 phase and directly binds to CtrA to prevent it from activating target genes. It forms a ternary complex with DNA and CtrA (CtrA:SciP:DNA complex) which protects SciP and CtrA from degradation by Lon and ClpXP respectively during G1 phase [34–59]. Cell cycle dependent regulation of another master regulator, CcrM is contributed by Lon protease [60]. CcrM, a DNA methyltransferase is present only in pre-divisional cells and is proteolyzed prior to cell division in *Caulobacter*. Lon null mutants have CcrM present throughout cell cycle despite the temporal regulation of transcription. This results in fully methylated chromosome throughout the cell cycle and eventually defects in morphogenesis and cell cycle progression.

**Table 3:** List of substrates degraded during cell cycle by Lon protease

Substrate	Role	Reference
SciP	Small CtrA inhibitory protein	34–59
CcrM	DNA methyltransferase	60
DnaA	DNA replication initiator	33
StaR	Transcriptional regulator of stalk biosynthesis	61
FliK	Flagella hook length regulator	61

Recently, novel substrates were identified that are subjected to Lon mediated proteolysis in *C. crescentus* as cell progresses through cell cycle. One is transcriptional regulator of stalk biosynthesis, StaR, and flagella hook length regulator, FliK [61]. StaR is a DNA binding protein involved in regulating stalk biosynthesis and holdfast development. Both these events need to be tightly regulated during cell cycle and proteolysis by Lon ensure the protein is present only when its function is required by clearing the protein as its transcription decreases in a cell cycle dependent manner. Also, the levels of FliK, that acts as a molecular ruler to determine the length of the flagella, are regulated by Lon protease such that it accumulates in late S phase where cell starts to prepare for division by building new flagellum at swarmer pole.

In addition, Lon has role in arresting the cell cycle during nutritional and proteotoxic stress by degrading an important cell cycle protein, DnaA [33]. Like many other bacteria, *Caulobacter* requires an active replication initiation factor DnaA, which is active in its ATP bound state and inactive in ADP bound state. During stress conditions such as accumulation of misfolded proteins, Lon protease rapidly proteolyzes DnaA leading to cell cycle arrest in G1 phase. This helps cells survive acute stress conditions and restore the normal homeostasis. Thus, Lon has, over time, emerged as an important protease in driving cell cycle and coordinating cell cycle event with cell differentiation.

### Regulatory proteolysis controls the levels of many cell division proteins

Cell division must be coordinated with other events of cell cycle like growth and replication of chromosomes. To divide or not is an important decision that cells take, taking into consideration the available resources and environmental conditions. In *Caulobacter crescentus*, different mechanisms including transcriptional regulation, in response to internal cues and external environmental cues, and regulatory proteolysis, control the cell division to ensure that cells divide at the right time and right place [62]. Owing to the stringent coupling between DNA replication and developmental state, the levels of many proteins change dramatically [37].

Divisome in bacteria include more than a dozen of proteins that are regulated in a cell cycle dependent manner [63, 64]. The components of this multiprotein complex assemble in a sequential manner at the midcell and drive the cytokinesis of cell by invaginating the inner and outer membranes as well as synthesizing and remodeling the peptidoglycan layer. The first protein recruited is an essential cell division protein, FtsZ, which forms a contractile Z ring at incipient site of division and acts as a scaffold for assembly of other cell division components [65, 66]. The establishment of midcell site is achieved through bipolar localization of MipZ [67], an inhibitor of FtsZ polymerization, with chromosomal segregation, which allows FtsZ to polymerize only at division site. Soon after FtsZ assembly, its early binding partners arrive to stabilize the FtsZ localization. Thereafter, factors responsible for peptidoglycan remodeling appear at mid cell followed by late arrival of FtsA, a protein that binds directly to FtsZ and stabilizes its tethering to the membrane. Consequent to FtsA appearance, five core divisome proteins arrive at division site with FtsQ mainly responsible for stabilizing the divisome. This initiates invagination of envelope followed by recruitment of polar markers. At this point cytoplasm begins to compartmentalize and finally the cell separates into two asymmetric daughter cells.

**Fig. 3:**
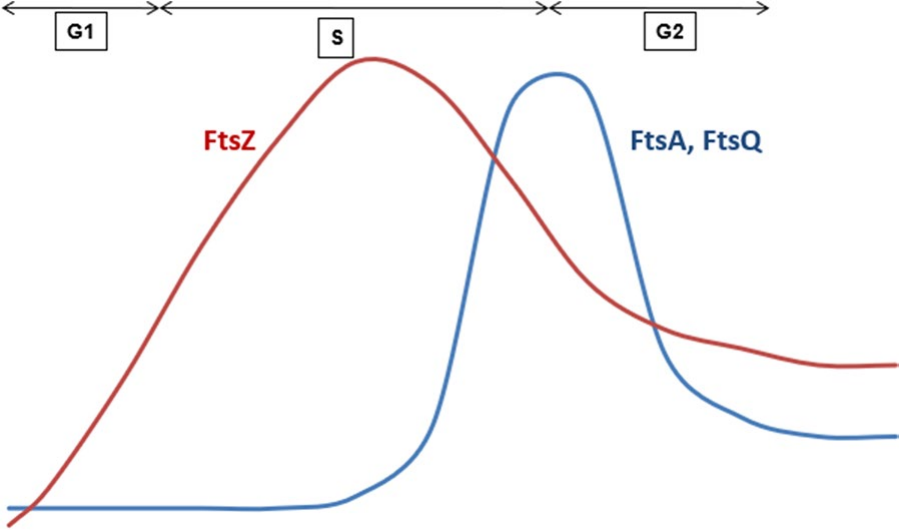
Graphical display of relative levels of cell division proteins FtsZ (red) and FtsA and FtsQ (blue) as cell progresses through cell cycle *: FtsZ levels peaks when the first signs of constriction begin and then decrease during pre-divisional phase. On the contrary, levels of FtsA/Q peaks in late predivisional cell and drops sharply after cytokinesis. *The points in graph are based on general observation of protein peaks during cell cycle

The synthesis/levels of FtsZ appear to be regulated differently in different bacteria [68]. While in *E.coli*, the concentration of FtsZ remains essentially constant throughout the cell cycle; its levels vary dramatically during the cell cycle of *Caulobacter crescentus*. The swarmer cells are devoid of FtsZ [Fig. 3]. The protein is first detected during the differentiation of non-replicating swarmer cell into replicating stalked cell and the peak levels are observed around the time when first signs of constriction become noticeable [69, 70]. The levels again decrease in predivisional cell once the constriction begins. After asymmetric division, only the stalked cell receives the little amount of FtsZ with swarmers receiving none of it. This change in concentration of FtsZ with the progression of cell cycle in *Crescentus* is regulated at transcriptional and post translational level in such a way that FtsZ is present in cell only when and where it is required for cell division. Temporal regulation of *ftsZ* transcription corresponds to the change of FtsZ levels and DNA replication as the cell proceeds through cell cycle. The key player involved in this is CtrA, the master regulator of transcription in *C.crescentus* [71]. CtrA in fact co-regulates FtsZ transcription with FtsA and FtsQ [72]. CtrA binds to *ctrA* box upstream of the *ftsZ* promoter and switches off the transcription of *ftsZ* gene in swarmer cells. As swarmer cell begins to differentiate into stalked cell, CtrA is specifically proteolysed by the cellular protease which relieves its inhibition on *ftsZ* transcription to kick off the synthesis of FtsZ. Proteolysis of CtrA is also seen in stalk precursor of late predivisional cell such that only stalked cells receive FtsZ after division.

In addition to the transcriptional control, the levels of FtsZ at different stages of cell cycle are regulated by proteolysis by means of ATP dependent ClpXP and ClpAP proteases in *Caulobacter* [69, 73]. ClpXP mediated degradation of FtsZ is conserved in other bacteria such as *E.Coli* where FtsZ and other cell division proteins are subjected to proteolysis by this protease [74]. In presence of Z-ring, ClpXP transiently localizes to mid-cell towards the end of cell cycle and might be the factor responsible for asymmetric distribution of FtsZ after cell separation where it is degraded in swarmer but not in the stalked daughter cell. The degradation is also linked to polymerization state of FtsZ and it is speculated that FtsZ is more stable in assembled state than in monomeric form [71]. This could be attributed to masking of recognition sites of proteases in polymeric form. This allows FtsZ to polymerise in stalked cells and form Z ring and but as cytokinetic ring constricts, FtsZ depolymerises unmasking the protease recognition sites and therefore its rapid degradation. In addition to FtsZ, regulatory proteolysis also controls the levels of other divisome components including FtsA and FtsQ [75]. While FtsA levels are regulated by ClpAP in vivo, the protease for FtsQ remains to be identified. The levels of both FtsA and FtsQ peaks in late predivisional cell and drops sharply after cytokinesis (Fig. 3). The degradation of these proteins during last stages of cell cycle allows coordination between cellular processes like cell division and DNA replication.

### Targeting regulatory proteolysis has applications in molecular medicine

The advent of genomic technologies together with the completion of the *Caulobacter crescentus* genome sequence have revolutionized the pace of research into the genetic networks that control the bacterial life cycle. Recent studies have shown that many of the mechanisms discovered in *Caulobacter* are evolutionarily conserved among other members of the α subdivision of proteobacteria including those having important roles in a wide range of environmental, medical and biowarfare protection applications. Therefore, the fundamental research on *Caulobacter* has far-reaching implications. The study of cell division proteins, identified previously [5], revealed that these proteins may be rapidly degraded regardless of the organism or proteolytic pathway involved because there is a universal need for the dynamic response. Although the precise details vary, the basic strategy for regulating cell cycle is conserved across many prokaryotic and eukaryotic organisms. Timely and regulated destruction of protein used by cells to control their growth and division, therefore, directly refers to the inference that dysregulation of proteolytic pathway can trigger a state of uncontrolled growth and proliferation in the cell.

Pathogenic bacteria rely on regulatory proteolysis for the virulence at multiple levels. From conferring resistance against stressful conditions in the host to regulating stability of virulence regulators, timely degradation of proteins by regulatory proteases ensure that the bacteria are able to thrive in the hostile host environment. The virulence of several pathogens has been seen to be implicated by ClpX and/or ClP [76]. In pneumonia causing *Staphylococcus aureus,* for instance, inactivation of proteases leads to attenuated virulence and stress sensitivity in bacteria in murine models [77]. Similarly, in food borne pathogen, *Listeria monocytogenes* [78], which is otherwise able of adapting of intracellular environment of host cells, loss of ClpP is shown to render the bacteria incapable of escaping phagocytosis and replication in host macrophages. In addition, the expression and activity of virulence factors is compromised in ClpP mutant pathogen. In *S. pneumoniae* also, deletion of ClpP affected the colonization of bacteria in lungs and reduced the mortality rate in infected murine models [79]. Regulatory proteases, thus, serve as promising targets against the pathogenecity. However, a deeper understanding of this regulation is required that could shed light on how cells respond to environmental cues in host organisms. Knowing how the novel identified players of regulatory proteolysis work at the molecular level can help figuring out how bacteria and other cells accurately distinguish waste from useful molecules. This could offer medical researchers a clue for controlling disease, such as bacterial infections.

A number of antibiotics have been developed previously that target regulatory proteases to control bacterial infection [80, 81]. Acyldepsipeptides, for example, kill bacteria by targeting ClpP and hampering its activity. Cyclomarin A is effective against tuberculosis as it binds to Clp protease of *Mycobacterium tuberculosis*, bacteria that is otherwise highly resistant to many antibiotics.

However, the recent alarming rise of multidrug-resistant bacteria has made vital the identification of new targets for antibiotics. Identification of proteins essential for bacterial survival like those involved in cell division and the mechanisms controlling them can be helpful in generation of new antibiotics. Research on proteases and mechanical insights into proteolysis can represent a promising area for clinical advances showing particular promise for the antibiotic therapies. The outcomes of the proposed research can help identify research areas and directions that will accelerate understanding protease biology and enhance clinical translation.

## Conclusions

Regulated proteolysis is essential for progression of cell through cell cycle. Loss of degradation leading to stabilization of the selected genes can interfere with cell cycle progression, cell division, DNA replication and cell growth. The study area and the results represent a fertile ground for advances that will be clinically useful e.g. drug development against bacterial invasions. Hence, highly conserved proteolytic systems in bacteria are excellent targets to elucidate therapeutic implications in bacterial infections.

## Data Availability

Not applicable.

## Abbreviations

Clp: Caseinolytic protease
MipZ: Midcell positioning of FtsZ
FtsZ: Filamenting temperature-sensitive mutant Z
FtsA: Filamenting temperature-sensitive mutant A
FtsQ: Filamenting temperature-sensitive mutant Q
CtrA: Cell cycle transcriptional regulator A
